# Beneficial effects of trehalose and gentiobiose on human sperm cryopreservation

**DOI:** 10.1371/journal.pone.0271210

**Published:** 2023-04-13

**Authors:** Dariush Gholami, Mohsen Sharafi, Vahid Esmaeili, Touba Nadri, Loghman Alaei, Gholamhossein Riazi, Abdolhossein Shahverdi

**Affiliations:** 1 Institute of Biochemistry and Biophysics (IBB), University of Tehran, Tehran, Iran; 2 Faculty of Biotechnology, Amol University of Special Modern Technologies, Amol, Iran; 3 Department of Embryology at Reproduction Biomedicine Research Center, Royan Institute for Reproductive Biomedicine, ACER, Tehran, Iran; 4 Department of Poultry Science, Faculty of Agriculture, Tarbiat Modares University, Tehran, Iran; 5 Department of Animal Science, Faculty of Agriculture, Urmia University, Urmia, Iran; 6 Department of Biological Science, Faculty of Science, University of Kurdistan, Sanandaj, Iran; University of Nebraska-Lincoln, UNITED STATES

## Abstract

The protection of human sperm during cryopreservation is of great importance to infertility. Recent studies have shown that this area is still a long way from its ultimate aim of maintaining the maximum viability of sperm in cryopreservation. The present study used trehalose and gentiobiose to prepare the human sperm freezing medium during the freezing-thawing. The freezing medium of sperm was prepared with these sugars, and the sperm were then cryopreserved. The viable cells, sperm motility parameters, sperm morphology, membrane integrity, apoptosis, acrosome integrity, DNA fragmentation, mitochondrial membrane potential, reactive oxygen radicals, and malondialdehyde concentration was evaluated using standard protocols. A higher percentage of the total and progressive motility, rate of viable sperm, cell membrane integrity, DNA and acrosome integrity, and mitochondrial membrane potential were observed in the two frozen treatment groups compared to the frozen control. The cells had less abnormal morphology due to treatment with the new freezing medium than the frozen control. The higher malondialdehyde and DNA fragmentation were significantly observed in the two frozen treatment groups than in the frozen control. According to the results of this study, the use of trehalose and gentiobiose in the sperm freezing medium is a suitable strategy for sperm freezing to improve its motion and cellular parameters.

## Introduction

The protection of human sperm during cryopreservation is of tremendous importance in normozoospermic individuals [1]. The cryopreservation process can increase oxidative stress and reactive oxygen species (ROS) in sperm, altering lipid and protein composition and decreasing sperm viability and motility [2]. One of the social concerns in young and adolescent people is maintaining their reproductive potential because they are at risk of injury or death (for example, members of the Armed Forces who are being deployed to a war zone). One way to improve infertility in these patients is sperm cryopreservation [3]. Cryopreservation has been improved to the outcomes of assisted reproductive technology programs [4], and preserved sperm in individuals facing medical treatment for a condition that may affect their fertility [5]. The individuals may want to consider freezing their sperm if they are a male transitioning to a female. They want to preserve their fertility using sperm cryopreservation before starting hormone therapy or reconstructive surgery [6]. Various sperm cryopreservation techniques, including vitrification [7], slow and fast cryopreservation [8], and several studies such as the use of cryoprotectants, cryopreservation protocols, and cryopreservation carriers have been reported [9].

Despite current sperm cryopreservation techniques, the concentrations of sperm maintaining normal post-thaw motility are still a matter of debate. The freezing process can damage sperm structure and function [10–12]. Therefore, the need for a new cryoprotectant or to improve existing ones is considered essential. One of the critical factors that can be effective is using various sugars in the sperm freezing medium [13]. Sugars play a crucial role during cryopreservation due to the interaction with the phospholipid membrane during dehydration conditions caused by the cryopreservation process [14]. Sugars can delay the membrane phase transition temperature in dehydrated lipids, and thus reduce the shedding of components and membrane fusion [15]. Therefore, some sugars such as glucose or fructose are used as an energy source for sperm during the freezing process. Some are intended to prevent the sperm from structural and sub-structural damage during this dehydrated state [16].

Trehalose and gentiobiose are two cryoprotectant agents that can be used. Trehalose is one of the cryoprotectants commonly used in the vitrification of sperm [17], but its role in the sperm fast freezing method appears to be unknown. It is also used to protect tubulin proteins in tubulin freeze-dried events [18]. In the presence of trehalose, dehydrated tubulin binds to the microtubule without conformational changes, and the microtubule is polymerized and fully functional [19]. The effects of gentiobiose on cryopreservation have not been studied so far, and this study addresses this issue. No studies have been conducted to evaluate the comparative effects of trehalose and gentiobiose on human sperm cryopreservation.

Then, gentiobiose cryoprotective effects on sperm in the presence and absence of trehalose are investigated. An attempt was made to use these two cryoprotectants to prepare a sperm cryopreservation medium. Therefore, it is possible to optimize a sperm freezing medium with higher cryopreservation effects than conventional cryogenic environments and to create a suitable substrate for maintaining sperm viability during the cryopreservation process.

This study aimed to use gentiobiose and trehalose as cryoprotectants to prepare the sperm freezing medium of human sperm during the freezing-thawing process.

## Material and methods

### Study population

Men of fertile couples referred for evaluation at an Infertility Center of Royan Research Institute in Iran have requested permission to discard semen samples for research purposes. After a verbal explanation, the written consent form or information sheet was obtained from all subjects and/or their legal guardian. All methods were carried out by the ethical guidelines of the Helsinki Declaration. The Research Ethics Committee approved the present study of Amol University of Special Modern Technologies, Amol, Iran (Ir.ausmt.rec.1400.20), and the Research Ethics Committee of Royan Institute, Tehran, Iran (http://ethics.research.ac.ir/IR.ACECR.ROYAN.REC.1397.033).

### Media preparation

Human sperm freezing medium was prepared as follows: 100 mM NaCl, 2.72 mM CaCl_2_, 0.492 mM MgCl_2_, 5.365 mM KCl, 30.95 mM NaHCO_3_, 0.32 mM NaH_2_PO_4_, 12.86 mM Sodium Lactate, 133.2 mM glycine, 20 mM Hepes-free acid, 15% V/V glycerol, 0.2 mM penicillin, and 0.1 mM streptomycin. Before adding trehalose and gentiobiose cryoprotectants to the freezing medium, they were first dissolved in distilled water and their aqueous solution was added to the freezing medium.

The Trehalose solution, including 0.025, 0.05, 0.1 and 0.2 M Trehalose were added to each medium, respectively (each medium was prepared with one concentration). Also, 0.025, 0.05, 0.1 and 0.2 M gentiobiose solution were separately added to each medium. Then, 200 μM Salidroside was added to the medium and stored at -20°C for the next experiment [20].

### Semen sample preparation

Semen samples were collected from 25 individuals with normal morphology >4%, motility >40%, and sperm concentration of >20×10^6^/ml according to world health organization (WHO) criteria [21] with 3–5 days of sexual abstinence.

This study was performed in a randomized design with four groups fresh control, frozen control, frozen trehalose, and frozen gentiobiose.

A non-programmable freezing technique has been used for sperm cryopreservation. The samples were liquefied at 37°C for 3 0 minutes and freezed using a new medium. Specimens were slowly mixed dropwise with an equal volume of each medium. The mixture was then loaded into labeled cryovials and placed in liquid nitrogen vapor on a tray 3 cm above the liquid nitrogen surface for 15 min before immersion in liquid nitrogen. The cryovials were immersed in liquid nitrogen for two or three weeks until further testing [22, 23].

Frozen-thawed samples were diluted four-fold with Tyrode’s salt solution. The diluted samples were centrifuged at 800 g for 4 min at 25°C. Then, the pellets were gently resuspended in 0.5 mL of Tyrode’s salt solution. The post-thaw sperm suspension was incubated at 37 ˚C in 5% CO2 for 20 min, followed by sperm analysis.

### Dose determination of trehalose and gentiobiose

To determine the optimal dose of trehalose and gentiobiose, concentrations of 0.025, 0.05, 0.1, and 0.2 M trehalose or gentiobiose were used to prepare a freezing medium. About 5% of human serum albumin (HAS) was added for each concentration. Then, sperm cells were frozen in the presence of a freezing medium prepared with these doses, and after the thawing process, the total motility of all cells was evaluated with a computer-assisted sperm analysis (CASA) (Version 5.1; Microptic, Barcelona, Spain) and the optimal dose for each combination was identified.

### CASA analysis

Total and progressive motility, the parameters of curvilinear velocity (VCL), straight linear velocity (VSL), average path velocity (VAP), linearity (LIN), straightness (STR), amplitude the of lateral head displacement (ALH), and beat-cross frequency (BCF) was assessed using a CASA system. For CASA analysis, 5 μl of the semen in all experimental groups were loaded onto a pre-warmed 20 μm chamber (Leja 4, Leja Products Luzernestraat B.V., Holland), placed on a warm slide at 37°C and analyzed by CASA system.

### Viability assessment

Sperm viability was assessed by Eosin-Nigrosin staining as described by Bjorndahl et al. [24]. Sperm smears were prepared by mixing equal volumes of the semen and Eosin-Nigrosin staining solution. The viability was assessed by counting 200 cells using a light microscope at 100× magnification. Colorless sperm and completely or partially stained sperm were considered live and dead sperm, respectively.

### Abnormal morphology

Abnormal morphology was evaluated by Papanicolaou staining. First, 20 μl of samples were placed on a slide and air-dried. The smears were then manually stained by Papanicolaou staining. Three hundred sperm were counted for each sample, and percentages of head and acrosome abnormalities were recorded by light microscopy at 100× magnification.

### Membrane integrity

Sperm membrane integrity was assessed by the hypo-osmotic swelling (HOS) test according to WHO criteria [21]. Semen was incubated with HOS (0.735 gr of sodium citrate dihydrate and 1.351gr of D-fructose were added in 100 ml of distilled water) medium at 37°C for 30 min, then 10 μl of the mixture with 10 μl of eosin stain was placed on a warm slide and membrane integrity percentage were measured by counting 200 cells by light microscope (400× magnifications, CKX41, Olympus, Tokyo, Japan) at 100× magnification. Sperm with swollen tails were considered live.

### Acrosome integrity

The acrosome integrity was assessed using the fluorescein conjugated lectin Pisum sativum agglutinin (FITC-PSA) staining protocol [21]. The specimen was smeared on the microscope slide, dried, and fixed in ethanol at 20°C for 30 min. The smear was stained with FITC-PSA and incubated at 4°C for 60 min. The number of 200 cells was evaluated in each replicate using fluorescence microscopy at 100× magnification at 450–490 nm excitation.

### DNA fragmentation determination

DNA fragmentation was determined using the acridine orange test (AOT) as described by Chohan et al. [25] with some modifications. A suspension of 30 μl of spermatozoa (10×10^6^ sperm/ml) was smeared onto a pre-cleaned glass slide, dried, and fixed in Carnoy’s solution (methanol /acetic acid, 3:1) at 4°C for 10 min. PBS first performed washing and then followed by 40 ml of citric acid solution (0.1 M) and 2.5 ml of Na2HPO47H2O (0.3 M) for 5 minutes. Finally, Acridine Orang aqueous solution (1%) was added, and the smear was subjected to fluorescence microscopy at 100 magnifications, with an excitation wavelength of 450–490 nm for 200 cells. Single-stranded DNA was observed as orange and double-stranded DNA was observed as green. DNA fragmentation index (DFI) was calculated according to the following equation: $\%DFI=\frac{red\;fluorescence}{total\;(red+green)\;fluorescence}\times 10^{2}$

### Evaluation of malondialdehyde concentration

Malondialdehyde (MDA) concentration was measured by the thiobarbituric acid (TBA) method [26, 27] with some modifications. The semen samples were first centrifuged at 1500g for 5 min. Then, sperm pellet and 100 μl of suspension as seminal plasma were separated to measure the level of MDA. For the measurement of seminal plasma, about 100 μl of suspension was mixed with 900 μl distilled water and 500 μl of TBA reagent (0.67 gr of thiobarbituric acid-2 in 100 ml of distilled water) then dissolved in 0.5gr NaOH. Afterward, 100 ml glacial acetic acid was slowly added to the vicinity of ice and then kept a 95°C for 30 minutes and cooled on ice for 5 minutes. The pellets were again centrifuged at 1500 gr for 5 min, and the supernatant absorbance was read by spectrophotometer at 534 nm, using an extinction coefficient of 1.56 × 10^5^ mol^-1^. Lcm^-1^ according to the A = εdc equation and reported in nmol/ml.

### Free radical assessment

The free radical level was assessed by the chemiluminescence assay using luminol (5-amino-2, 3-dihydro-1, 4-phthalazinedione; Sigma, USA) dissolved in dimethyl sulfoxide as a probe. First, 10 μl luminol working solution (5 mM) was added to 400 μl liquefied sperm suspension (20×10^6^ sperm/ml) and mixed slowly.

Semen was centrifuged with PBS for 7 min, and a mixture of 20 × 10^6^ sperm/ml was then prepared by PBS. 395 μl PBS, 5 μl 30% H_2_O_2_ and 10 ul luminol working solution were designed as positive controls. Also 400 μl PBS and 10 μl luminol working solution were designed as negative controls.

Chemiluminescence was performed for 15 min using a luminometer and results were recorded as RLU/Sec/20×10^6^ sperm.

### Total antioxidant capacity

Total antioxidant capacity was assessed using the total antioxidant capacity (TAC) assay kit (catalog # JM-K274-100). Accordingly, first lyophilized Torolox was dissolved in 20 μl of pure DMSO and then 980 μl of distilled water was added. 1 mM solution was prepared and maintained at -20°C for 4 months. To prepare the standards, concentrations of 0, 4, 8, 12, 16 and 20 μl distilled water were first removed, and Torolox was added instead. One portion of the copper reagent was added to 49 portions of the diluents assay to prepare the working solution. Thawed semen samples of control and experimental groups were diluted up to 20 times with distilled water. Afterward, 100 μl of the working solution was added to each well of the plate and incubated at room temperature for 90 minutes. The absorbance of the formed colored complex was recorded using the plate reader at 570 nm.

### Evaluation of mitochondrial membrane potential

A 1mL aliquot of semen (3×10^6^ sperm/ml) was stained with 1.0 μl of 1.53 mM 5,5′,6,6′-tetrachloro-1,1′3,3′-tetrathylbenzimidazolyl-carbocyanine iodide (JC-1) stock solution for 15 minutes at 37°C and centrifuged for 5 minutes at 800 g to determine mitochondrial membrane potential. The pellet was diluted at 1:5 in PBS and instantly evaluated for orange and green staining by flow cytometry.

For analysis of each specimen, a total of 10,000 gated events based on the FS and SS have been interpreted per specimen utilizing the flow cytometer. A 488 nm filter was applied for excitation of JC-1, and emission filters of 530 and 575 nm were used to quantify the community of spermatozoa with green and red fluorescence, respectively. FL1 and FL2 canals recognized sperm with JC-1 staining. The percentage of the spermatozoa population with red fluorescence indicates the high mitochondrial membrane potential. Also, the percentage of spermatozoa population with green fluorescence shows a low mitochondrial membrane potential. Therefore, the high proportion of red to green (red/green ratio) indicates a higher membrane potential.

### Assessment of apoptosis in spermatozoa

A detection kit (IQ Products BV, Rozenberglaan 13a 9227 DL Groningen, the Netherlands) used Annexin V to detect apoptotic cells. Specimens were washed in 1 ml calcium buffer 1X and centrifuged at 800 g for 5 minutes. A 10 μl of Annexin V FITC was added to the pellet (1 × 10^6^ sperm/ml) and incubated for 20 minutes at 4°C. The 10 μl propidium iodide (PI) was then added to the cell suspension and incubated for 10 minutes at 4°C. The stained cells were analyzed by flow cytometry at the fluorescence emission at 530 nm in the FL1 canal and 575 nm in the FL3 channel. We classified the normal sperm as (An^-^/PI^-^), early apoptotic sperm as (An^+^/PI^-^), apoptotic sperm as (An^+^/PI^+^), and necrotic sperm as (An^-^/PI^+^).

### Statistical analysis

Data were analyzed by SPSS software version 16 (SPSS Inc. Chicago, ILL). The statistical analysis was performed by one-way ANOVA followed by Tukey post hoc analysis. In all experiments, P-values less than 0.05 were regarded as statistically significant.

## Results

### Dosage of trehalose and gentiobiose

**Fig 1:**
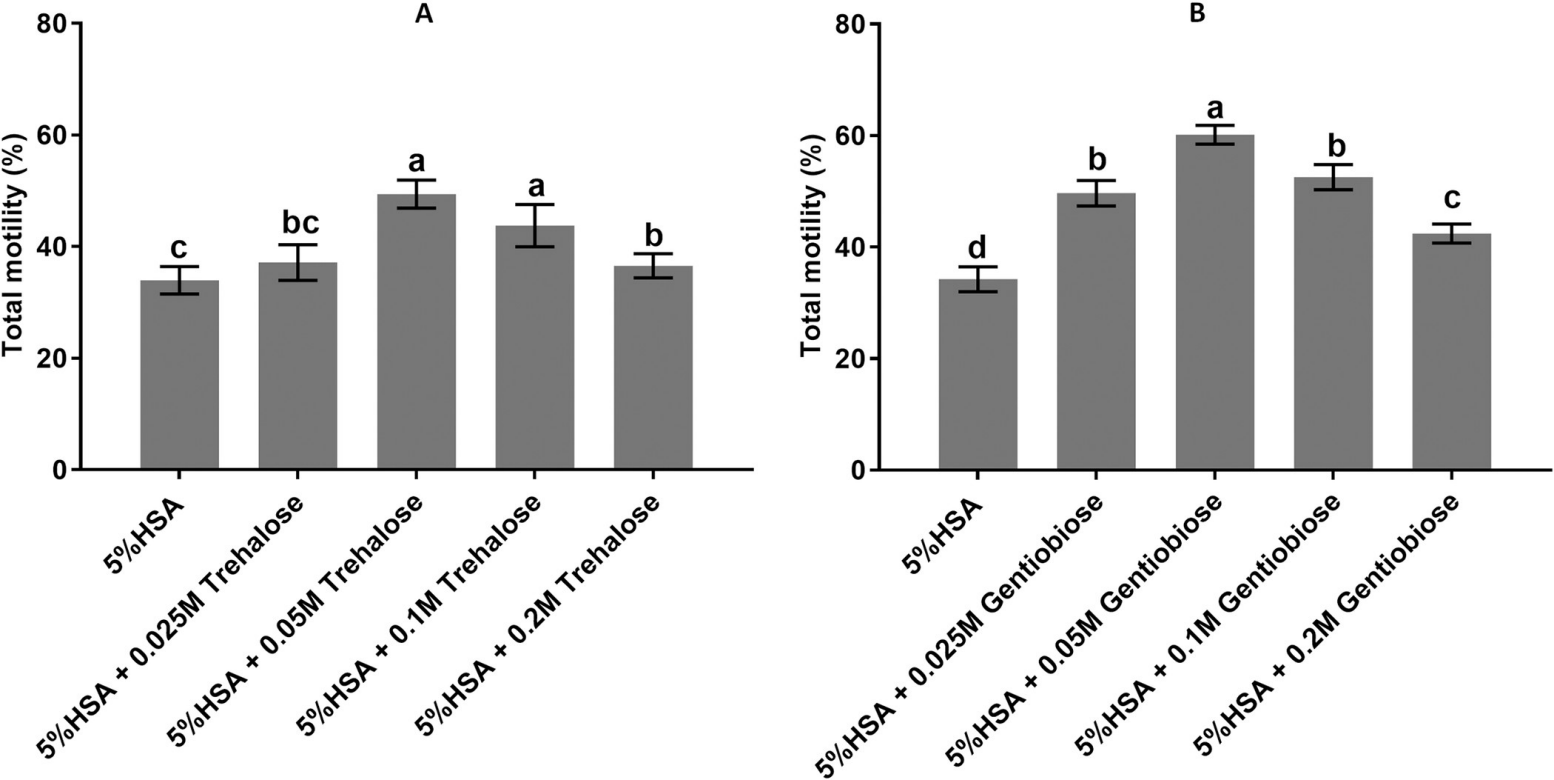
Dosage of trehalose and gentiobiose. The concentration of 0.05M of trehalose is presented as the highest total motility among the other concentrations (A). The concentration of 0.05M of gentiobiose is shown as the most increased total motility among the different concentrations (B). Values are expressed as mean ± SD, Tukey test; n = 25.

Fig 1 and S1 Table in S1 File show the percentage in different doses of trehalose and gentiobiose. The dose of 0.05M has the highest total motility in different concentrations of trehalose and gentiobiose (p <0.05, 49.4 ± 1.67%). Therefore, a concentration of 0.05M was selected as the optimal concentration for trehalose and gentiobiose and used in the following experiments.

### CASA analysis

CASA analysis is a semen analysis to screen for male fertility problems. This automatic system evaluates the concentration, motility and morphology of sperm. CASA systems have evolved through advances in microscope imaging devices, massive increases in computing power, new computer languages, and upgraded software algorithms.

Post-thaw sperm parameters are represented after the cryopreservation in all experimental groups as mean ± SD in Table 1 & S2 Table in S1 File. Total and progressive motility were significantly lower in frozen control (P < 0.05) than in other experimental groups. These parameters were significantly lower in frozen trehalose than in frozen gentiobiose (P < 0.05). The non-progressive motility was not significantly different among the experimental groups (P > 0.05).

**Table 1:** The CASA analysis.

Characteristics	Groups			
	Fresh Control (n = 25)	Frozen Control (n = 25)	Frozen Trehalose (n = 25)	Frozen Gentiobiose (n = 25)
Total motility (%)	82.93 ± 6.14^a^	46.84 ± 2.51^d^	58.44 ± 3.89^c^	68.66 ± 32.86^b^
Progressive (%)	61.32 ± 11.08^a^	21.95 ± 6.56^d^	30.51 ± 9.80^c^	41.35 ± 12.9^b^
Non-progressive (%)	22.23 ± 8.72	24.88 ± 6.57	27.52 ± 9.75	27.25 ± 11.72
**Motion variables**				
VCL (μm/s)	84.74 ± 9.99^a^	49.84 ± 16.33^c^	60.27 ± 19.4^bc^	66.12 ± 17.56^b^
VSL (μm/s)	44.51 ± 15.8^a^	22.31 ± 7.98^c^	27.13 ± 9.83^bc^	35.65 ± 12.17^b^
VAP (μm/s)	58.76 ± 18.31^a^	27.17 ± 11.43^c^	33.01 ± 12.84^c^	46.13 ± 10.15^b^
LIN (%)	49.38 ± 8.89^a^	32.08 ± 13.01^b^	40.2 ± 13.9^b^	46.13 ± 7.99^ab^
STR (%)	74.5 ± 6.9^a^	64.02 ± 14.94^b^	67.58 ± 14.57^ab^	74.36 ± 6.9^a^
ALH (μM)	2.36 ± 0.6^a^	1.58 ± 0.56^c^	1.76 ± 0.43^bc^	2.07 ± 0.41^ab^
BCF (Hz)	15.29 ± 4.2^a^	9.67 ± 2.34^c^	10.13 ± 3. 2^c^	12.44 ± 1.24^b^

Total and progressive motility and sperm motion variables

The assigned letters a, b, c, and d indicate significant differences (P < 0.05)

Values are expressed as mean ± SD, Tukey test

VCL, curvilinear velocity

VSL, straight linear velocity

VAP, average path velocity

LIN, linearity

STR, straightness

ALH, the amplitude of lateral head displacement

BCF, beat-cross frequency.

The VCL and VSL were significantly (P < 0.05) higher in frozen gentiobiose than in frozen control. There was no significant difference between frozen gentiobiose and frozen trehalose. The VAP parameter was significantly (P < 0.05) higher in frozen gentiobiose than in other frozen groups. This parameter showed no difference between frozen trehalose and frozen control (P > 0.05).

The LIN parameter was significantly (P < 0.05) higher in fresh control than in frozen control and frozen trehalose. At the same time, there was no significant difference between fresh control and frozen gentiobiose. The LIN parameter did not significantly differ between frozen groups (P > 0.05).

The STR was significantly (P < 0.05) higher in frozen gentiobiose than in frozen control. However, the difference in STR parameter between fresh control, frozen gentiobiose and frozen trehalose was not significant (P > 0.05).

The ALH parameter was significantly higher in frozen gentiobiose than in frozen control. This parameter revealed no difference between frozen gentiobiose and frozen trehalose (P > 0.05). Moreover, the BCF parameter was significantly higher in frozen gentiobiose than in frozen control and frozen trehalose (P < 0.05).

### Viability assessment

**Fig 2:**
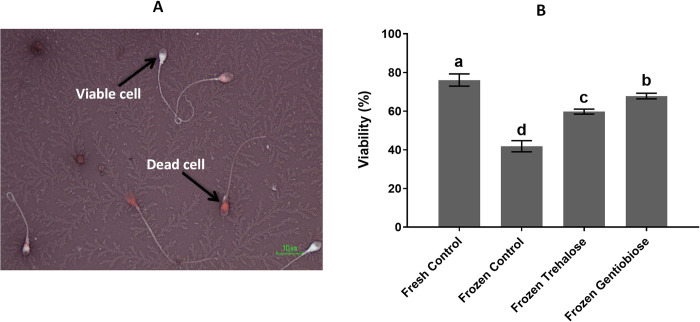
The viability of sperm. The viable and dead cells are observed in panel A. The Eosin-Nigrosin staining was used, where colorless sperm and complete/partial stained sperm show live and dead sperm, respectively (A). The percentage of viable cells differed in all experimental groups (B). The assigned letters a, b, c, and d indicate significant differences (P < 0.05). Values are expressed as mean ± SD, Tukey test; n = 25.

Sperm viability Assessment is essential for semen analysis in human reproductive medicine. Fig 2 and S3 Table in S1 File show the percentage of sperm viability in all experimental groups. There was a significantly lower percentage of viability in frozen control (41.98 ± 2.79%) than that in fresh control (75.14 ± 3.12%), frozen trehalose (58.61 ± 2.59%) and frozen gentiobiose (66.51 ± 1.39%; p< 0.05). A higher percentage of sperm viability was significantly (P < 0.05) observed in frozen gentiobiose compared to other frozen groups.

### Abnormal morphology

**Fig 3:**
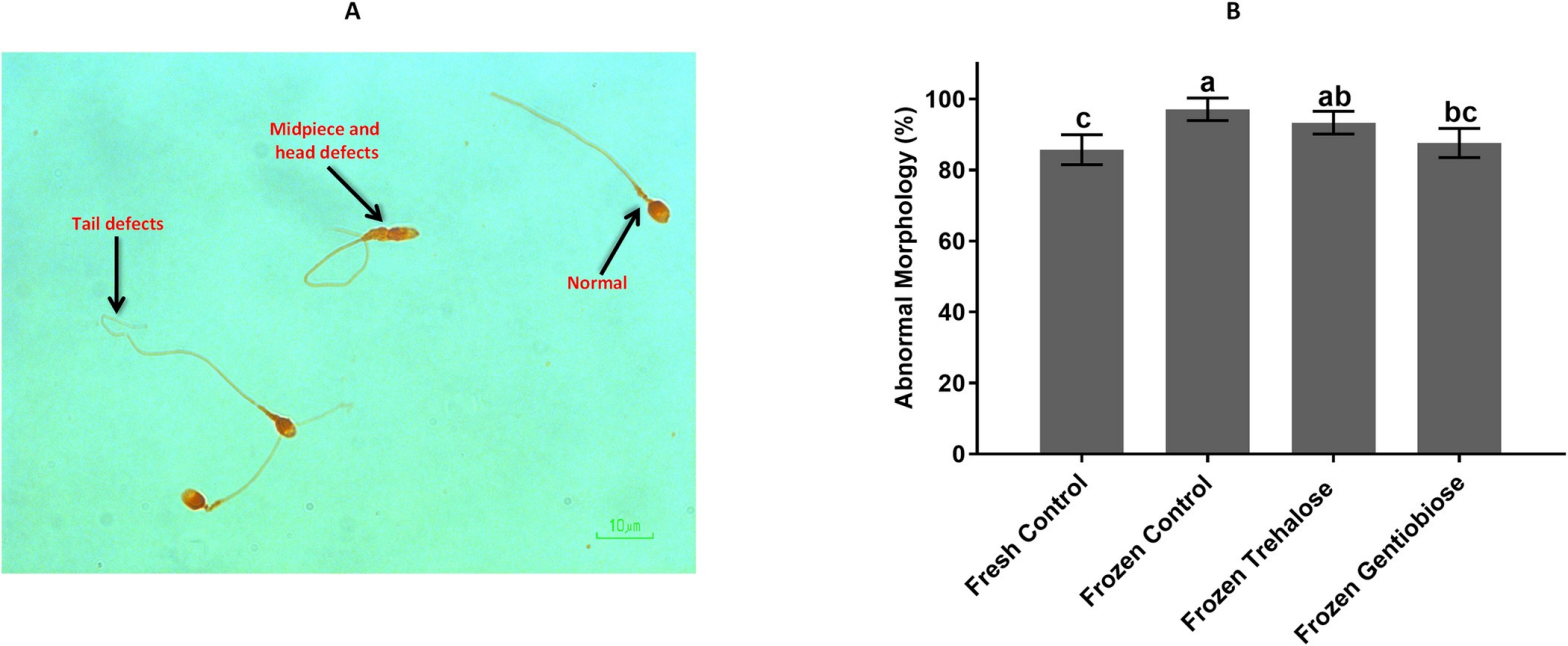
Sperm morphology. Normal and abnormal morphology of sperm as indicated by Papanicolaou staining; the intact cells and defects of the tail, head, and midpiece in sperm are observed (A), and the percentage of abnormal morphology differs in all of the groups (B). The assigned letters a, b and c indicate significant differences (P < 0.05). Values are expressed as mean ± SD, Tukey test; n = 25.

Abnormal morphology of sperm has defects in the head or tail such as a large or deformed head or a crooked or double tail. These defects may affect the ability of sperm to reach and penetrate the egg. Therefore, evaluating sperm morphology during cryopreservation is of great importance. As shown in Fig 3 and S4 Table in S1 File, the average abnormal morphology was significantly (p < 0.05) lower in frozen gentiobiose (87.64 ± 4.1%) as compared to frozen control and frozen trehalose (97.1 ± 3.17, and 93.32 ± 3.2%, respectively). This value was no significant difference between fresh control and frozen gentiobiose (P > 0.05).

### Membrane integrity

**Fig 4:**
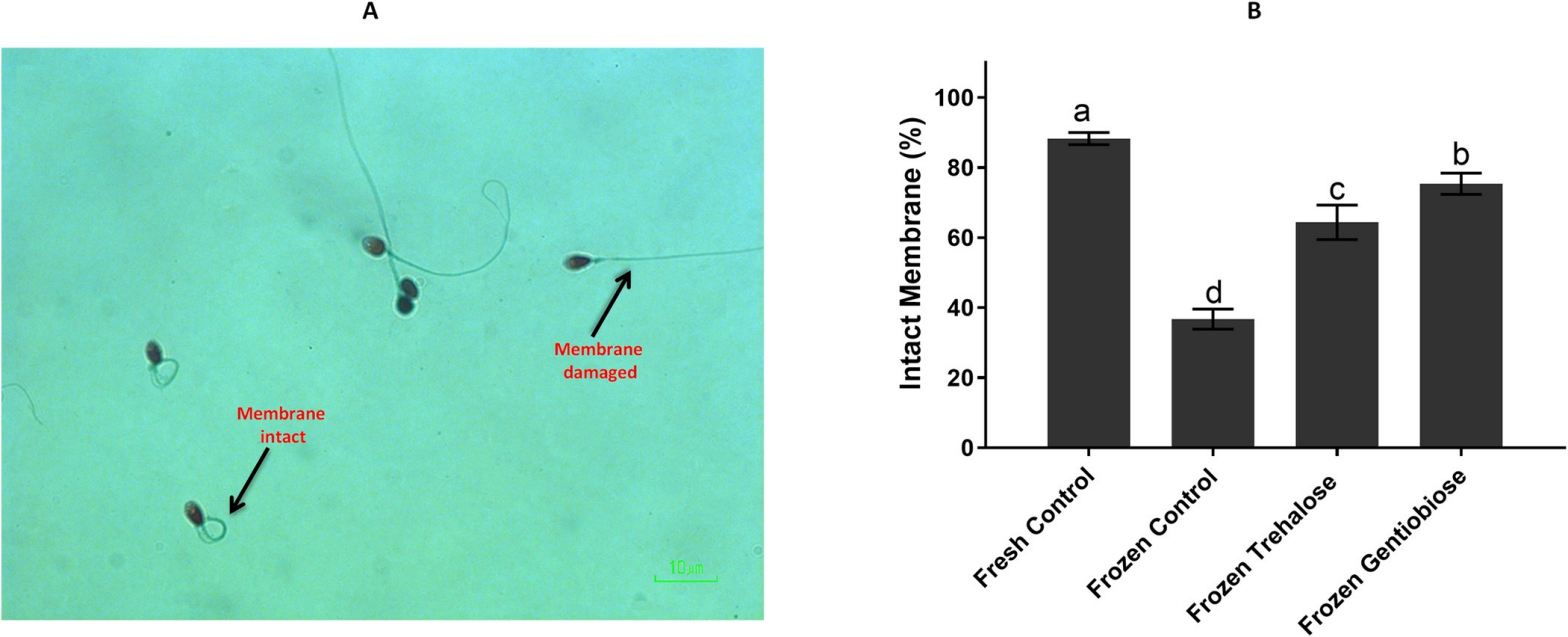
Membrane integrity of sperm was estimated using the HOS test. The membrane intact and damaged are observed (A), and the percentage of membrane-intact differs among the experimental groups (B). The assigned letters a, b, c, and d indicate significant differences (P < 0.05). Values are expressed as mean ± SD, Tukey test; n = 25.

Plasma membrane integrity is crucial for sperm survival within the female reproductive system to maintain fertilization capacity and osmotic balance of the cell and acts as a barrier between intra- and extracellular media. As shown in Fig 4 and S5 Table in S1 File, the membrane integrity was significantly (p < 0.05) lower in frozen control (36.76 ± 2.86%) than that in fresh control (88.24 ± 1.71%), frozen trehalose (64.4 ± 4.93%) and frozen gentiobiose (75.36 ± 3.05%). In addition, this value was significantly (p < 0.05) lower in frozen trehalose than that in frozen gentiobiose.

### Acrosome integrity

**Fig 5:**
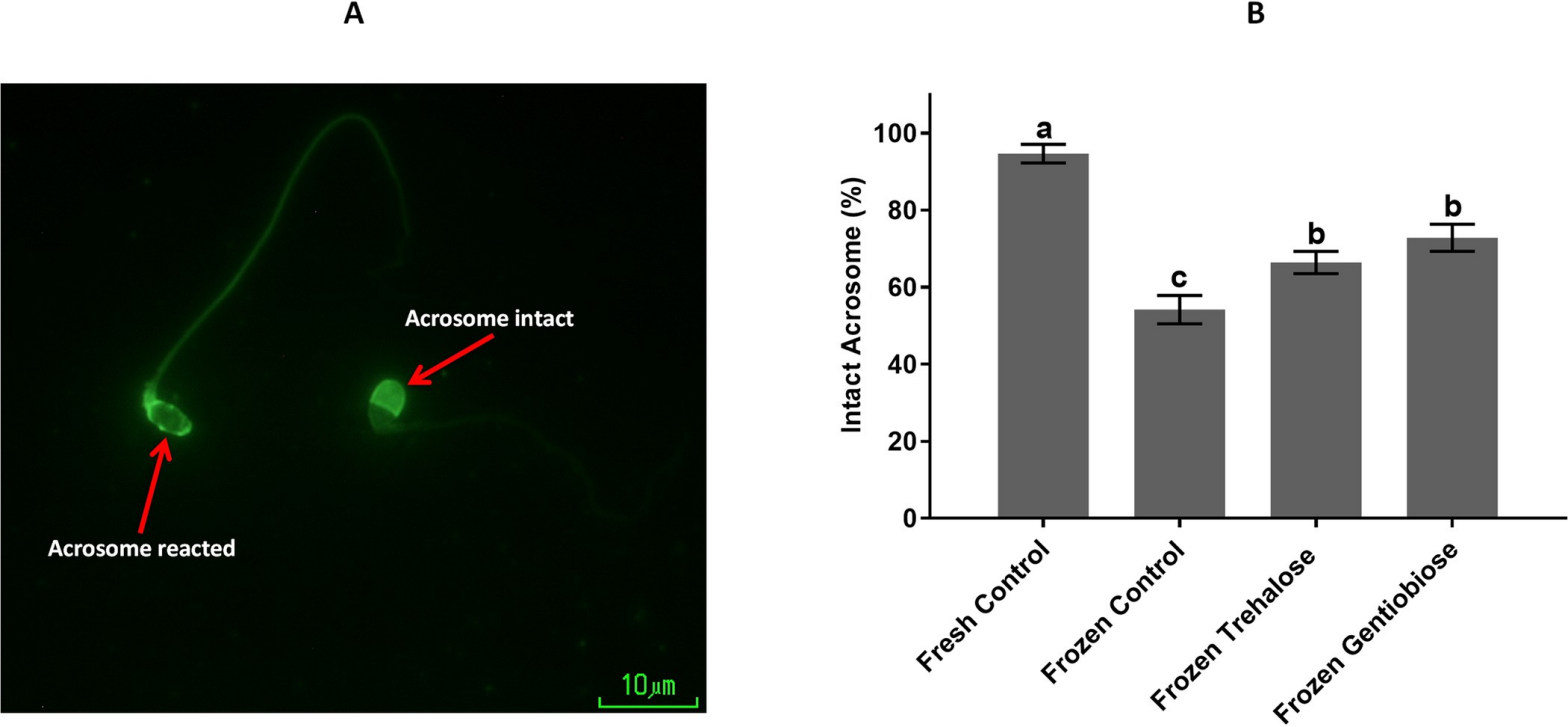
The acrosome integrity was assessed using the fluorescein conjugated lectin Pisum sativum agglutinin (FITC-PSA) staining. Sperm with intact and reacted acrosome was observed (A), and the percentage of intact acrosome significantly differs in all groups (B). The assigned letters a, b and c indicate significant differences (P < 0.05). Values are expressed as mean ± SD, Tukey test; n = 25.

A possible alternative to assessing sperm function is to evaluate the acrosome reaction, an important event for sperm fertilization. The acrosome is a large secretory granule that contains several hydrolytic enzymes that release these hydrolytic enzymes, destroying the zona pellucida and allowing sperm to penetrate it and attach to the egg in a process called acrosome reaction. Acrosome integrity is expressed as the percentage of sperm cells with normal acrosome morphology. As shown in Fig 5 and S6 Table in S1 File, the acrosome integrity was significantly (p < 0.05) lower in frozen control (54.19 ± 3.69%) than that in fresh control (94.66 ± 2.44%), frozen trehalose (66.4 ± 4.23%) and frozen gentiobiose (72.84 ± 3.51%). In addition, this value was (p < 0.05) lower in frozen trehalose than that in frozen gentiobiose.

### DNA fragmentation index

**Fig 6:**
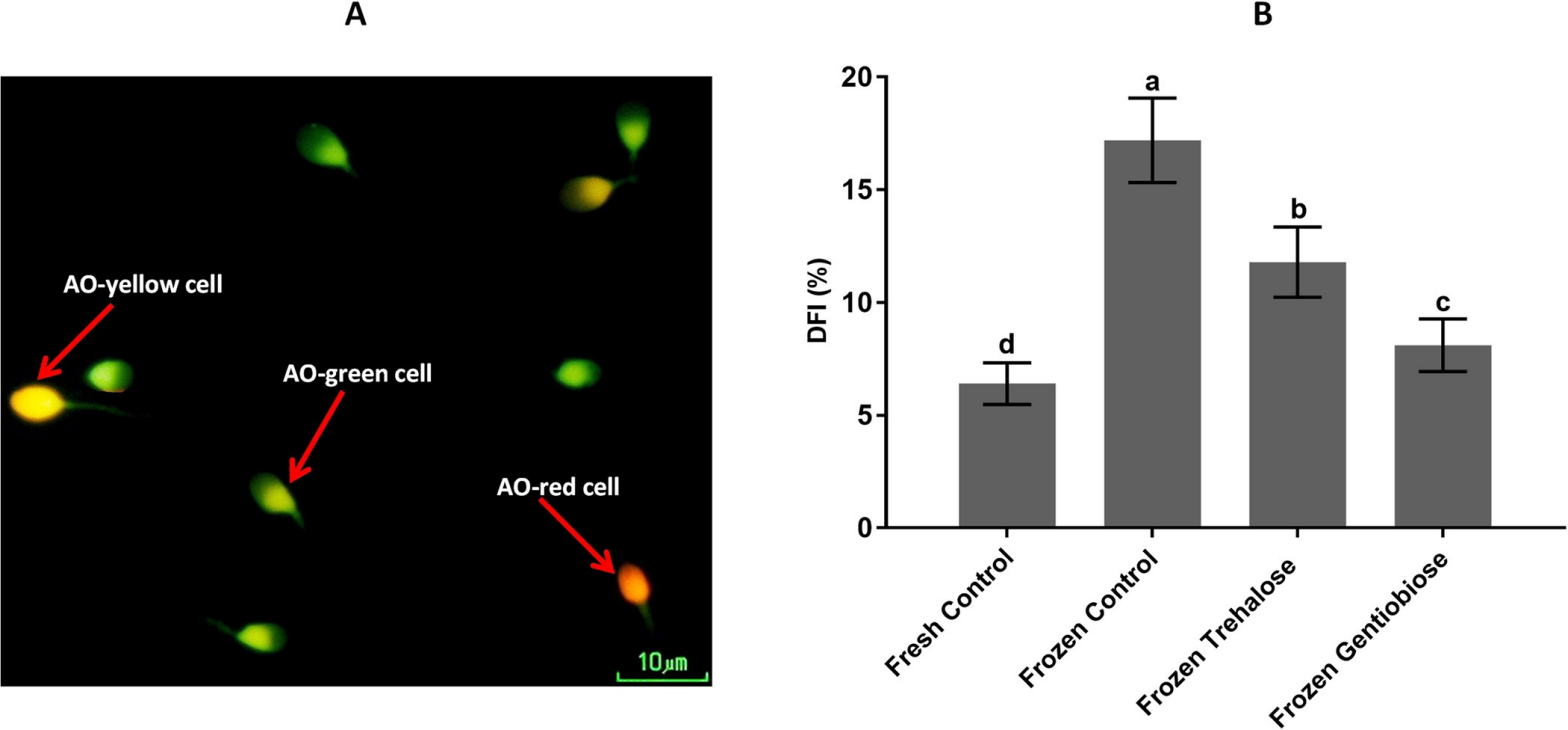
DNA fragmentation index was determined using AOT. Intact DNA is shown as green cells, and damaged DNA is observed as yellow and red cells by fluorescent microscopy with 100× magnification (A). The DFI was significantly different in all of the groups (B). The assigned letters a, b, c, and d indicate significant differences (P < 0.05). Values are expressed as mean ± SD, Tukey test; n = 25.

Sperm DFI is a genetic assessment that reflects the DNA damage and integrity, thus identifying potential sperm damage. It is an important indicator in evaluating semen quality. The DFI is shown in Fig 6 and S7 Table in S1 File. The highest rate of DFI was seen in frozen control (17.18 ± 1.87%, p < 0.05). Also, this index was (p < 0.05) higher in frozen trehalose (11.77 ± 1.56%) than frozen gentiobiose (8.06 ± 1.16%).

### The malondialdehyde, ROS, and total antioxidant capacity assessments

Sperm are sensitive to oxidative damage, and this abnormality can be measured by diagnostic tests, including lipid peroxidation, ROS, and TAC.

As shown in Table 2 and S8 Table in S1 File, the highest malondialdehyde concentration and ROS level were significantly (p < 0.05) observed in the frozen control. However, the lowest TAC was shown considerably in the frozen control. These three indicators did not significantly differ between frozen trehalose and frozen gentiobiose (p > 0.05).

**Table 2:** Comparison of MDA, ROS, TAC and mitochondrial membrane potential among the experimental groups.

Characteristics	Groups			
	Fresh Control (n = 25)	Frozen Control (n = 25)	Frozen Trehalose (n = 25)	Frozen Gentiobiose (n = 25)
Seminal MDA (nmol/ml)	31.31 ± 0.39^c^	45.78 ± 3.34^a^	35.78 ± 2.7^b^	34.12 ± 2.43^b^
Seminal ROS (RLU/Sec/20×10^6^sperm)	532.60 ± 4.77^c^	1107.74 ± 12.03^a^	838.64 ± 6.61^b^	751.56 ± 10.71^b^
Seminal TAC (nmol/μl)	19.13 ± 0.76^a^	5.42 ± 0.82^c^	13.62 ± 0.8^b^	14.3 ± 0.53^b^
Mitochondrial membrane potential (Read/Green ratio)	1.97± 0.09^a^	0.39 ± 0.02^d^	0.68 ± 0.06^c^	1.35 ± 0.16^b^

The assigned letters a, b, c, and d indicate significant differences (P < 0.05)

Values are expressed as mean ± SD

Tukey test; n = 25.

### Evaluation of mitochondrial membrane potential

The proton concentration and the electrical potential across the mitochondrial membrane lead to energy storage in the mitochondria. The electron transfer chain creates the proton gradient in the inner mitochondrial membrane. The penetration of lipophilic cations into the inner mitochondrial membrane causes the negative inner membrane potential. Therefore, mitochondrial function analysis may provide a way to detect sperm motility and assess motility levels as a direct indicator of viability and fertilization ability. Measuring the inner mitochondrial membrane potential (MMP) can easily determine sperm viability. Mitochondrial energy status is indicated by the MMP, which regulates intact functional mitochondria and is directly related to sperm motility.

**Fig 7:**
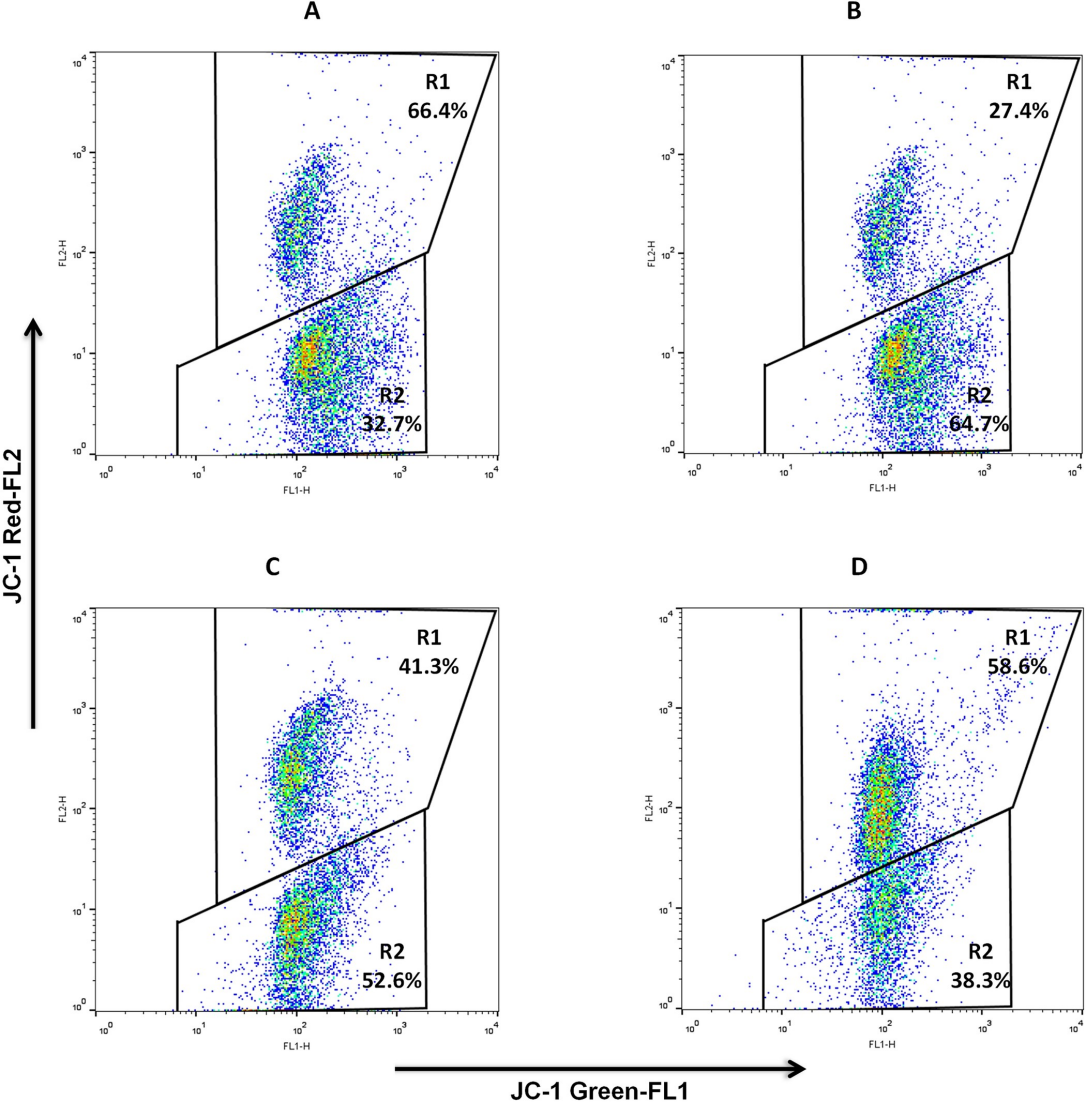
Evaluation of mitochondrial membrane potential. The panel of flow cytometry was stained with JC-1 for fresh control (A), frozen control (B), frozen trehalose (C), and frozen gentiobiose (D). The top right quadrant represents the percentage of values showing the proportion of sperm with high mitochondrial membrane potential. The bottom right quadrant represents the percentage of values that display the proportion of sperm with low mitochondrial membrane potential.

Fig 7, Table 2, and S8 Table in S1 File show that the frozen control observed the lowest red/green fluorescence ratio (P < 0.05). This ratio was significantly (P < 0.05) decreased in frozen trehalose and frozen gentiobiose compared to fresh control. Moreover, this ratio was significantly (p < 0.05) lower in frozen trehalose than in frozen gentiobiose.

### Assessment of apoptosis in spermatozoa

**Fig 8:**
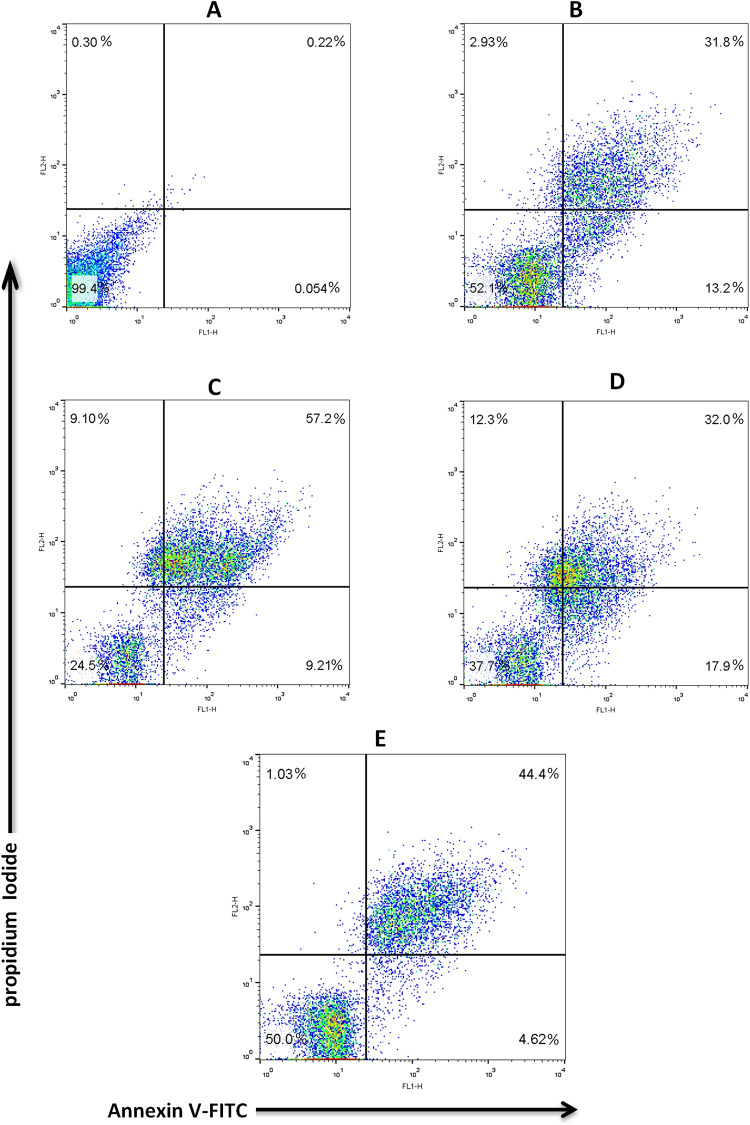
The sperm apoptosis in the cryopreservation process was assessed using Annexin V FITC and PI. Unstained sperm (panel A), a fresh control panel (B), a frozen control (panel C), Frozen trehalose (panel D), and a frozen gentiobiose (panel E) are presented in the graph. The top left quadrant shows necrotic cells; the lower left quadrant shows live cells; the lower right quadrant and the top right quadrant represent early apoptotic and late apoptotic cells, respectively.

Apoptosis is the usual mechanism of germ cell death during normal spermatogenesis and has been considerably studied in spermatogonia, spermatocytes, and spermatids. The loss of phospholipid asymmetry, which results in exposure to phosphatidylserine on the outer surface of the plasma membrane, is an early event of apoptosis in all tested human cells. Annexin V anticoagulants preferentially bind to negatively charged phospholipids such as phosphatidylserine. During apoptosis, cells attach to Annexin V before the plasma membrane loses its ability to remove PI. Therefore, by staining the cells with a combination of Annexin V and PI, the population of live sperm, apoptosis and necrosis can be detected simultaneously. As shown in Fig 8, Table 3 and S9 Table in S1 File the populations of live sperm were significantly (p < 0.05) higher in frozen gentiobiose than in frozen trehalose and frozen control.

**Table 3:** Sperm staining by Annexin V and PI.

Sample	N	An^-^/PI^-^ (%)	An^+^/PI^-^ (%)	An^+^/PI^+^ (%)	An^-^/PI^+^ (%)
**Fresh Control**	25	59.51 ± 2.33^a^	12.27 ± 1.42^b^	23.58 ± 3.44^c^	4.62 ± 2.7^b^
**Frozen Control**	25	35.03 ± 2.23^c^	15.36 ± 3.00^a^	38.11 ± 2.07^a^	12.00 ± 2.8^a^
**Frozen Trehalose**	25	51.65 ± 1.64^b^	11.48 ± 2.9^b^	27.27 ± 1.23^b^	9.58 ± 3.5^ab^
**Frozen Gentiobiose**	25	57.5 ± 3.66^a^	9.8 ± 4.0^b^	23.65 ± 1.61^b^	9.14 ± 3.5^b^

The assigned letters a, b, c, and d indicate significant differences (P < 0.05)

Values are mean ± SD

Tukey test. An^-^/PI^-^, live cells; An^+^/PI^-^, early apoptotic cells; An^+^/PI^+^, late apoptotic cells; An^-^/PI^+^, necrotic cells.

The percentage of early apoptotic and late apoptotic cells was significantly (p < 0.05) higher in the frozen control than in the other experimental groups. These values were not significantly different between the frozen trehalose, and frozen gentiobiose.

The percentage of necrotic cells was significantly (P < 0.05) lower in frozen gentiobiose than in frozen control. However, there was no significant difference between frozen gentiobiose and frozen trehalose.

## Discussion

Despite sperm cryopreservation being widely used for fertility preservation, one should not ignore its detrimental effects such as motility malfunctions, reduction of viability rate, and other sperm parameter defects [22, 28]. Usage of compounds with antioxidant activity such as sugars during cryopreservation indicated positive effects on sperm parameters [29, 30]. Sugars play an essential role in protecting cells, organisms, and biomolecules, including proteins and lipid membranes [31]. Sugars carry out their bioprotective activities through several different mechanisms. The following can be mentioned: 1) The water substitution hypothesis suggests that sugars replace water molecules during dehydration and form hydrogen bonds with charged and polar groups on the surface of biological molecules. In this way, denaturation of proteins and transition to gel phase in membranes is prevented [32–34]. 2) The headgroup-bridging hypothesis suggests that sugars form a scaffold of hydrogen bonds bridging multiple headgroups that hinder a transition to the gel phase undergoing dehydration and protect the membrane [35]. 3) The vitrification hypothesis proposes that sugars in biological systems are excellent glass-forming agents that protect biological molecules by forming amorphous crystals. Thus, they prevent structural fluctuations, denaturation of proteins, and mechanical destruction of membranes [36, 37]. 4) The water entrapment hypothesis suggests that sugars surround the remaining water molecules close to the surface of the biomolecules, thereby preserving the surface of biomolecules from dissolving with water molecules and retaining their natural properties in the quasi-dry state [38, 39].

In the current study, cryopreservation in the thawing stage has harmful effects on sperm parameters. It reduces the motility and progressive movement of the sperm, which are similar to results obtained in previous studies [1, 22, 40]. Such damage can jeopardize the intracellular organs involved in sperm survival due to changes in osmotic pressure and the formation of ice crystals during the cryopreservation process [22, 41]. Therefore the cryopreservation process can cause ROS production [28, 41, 42]. We proposed using gentiobiose and trehalose as freezing medium supplementation to minimize the detrimental effects of the cryopreservation process. As these sugars are non-permeable cryoprotectants, they cover the outer layer surface of the sperm membrane, stabilize, and protect the cell membrane structure. Only a few studies have investigated the effects of trehalose on human sperm cryopreservation [17, 20, 43]. Moreover, to the best of our knowledge, no studies have been performed on the impact of gentiobiose on sperm cryopreservation. Our study reported that 0.05 M trehalose and gentiobiose have the best post-thawed sperm parameters among all doses. This indicates that the osmolarity of the sperm cryopreservation medium is critical for sperm parameters during cryopreservation. Higher or lower concentrations than 0.05 M trehalose and gentiobiose may cause the hyperosmolarity or hypoosmolarity of sperm, respectively, leading to adverse effects on sperm motility and survival parameters [13, 17]. In addition, sperm equilibration in sperm freezing medium supplemented with cryoprotectants led to increasing moderately osmolarity in the range of 350 up to 430 mOsm/kg. It was observed a stimulating effect of the medium. Further increase in cryoprotectants leads to a high concentration in osmolarity up to 600 mOsm/kg, with a slight decrease in motility (4.5%). However, the hyperosmolarity of the freezing medium reduced sperm viability down to 45%.

In this study, the measured osmolarity in 0.05M trehalose and gentiobiose is between 250 and 350 mM/l, which is the appreciated osmolarity of the sperm cryopreservation medium [43].

Trehalose might positively affect some sperm parameters, such as sperm count, percent motility, normal morphology, and vitality [20, 43]. So far, no study has been observed on the effects of gentiobiose on these parameters. Based on the data of this study, we suggest that gentiobiose also has a positive impact on these sperm parameters during cryopreservation. These positive effects could be due to the impact of these components on lowering malondialdehyde levels and increasing total antioxidant capacity [44–46].

In the present study, the seminal ROS and malondialdehyde concentrations were evaluated in frozen-thawed sperm and were reduced in the freezing medium supplemented with trehalose or gentiobiose. The previous studies were shown the detrimental effects of excessive ROS levels on sperm [1, 47]. Our finding is in line with the earlier studies, which claimed that the ROS levels were reduced in the presence of Crocin) crocetin di-gentiobiose ester) in cattle sperm, an antioxidant that scavenges free radicals such as superoxide anion [48, 49]. Also, trehalose has a protective effect on lipid peroxidation in bovine calf testicular tissues [50] and suppresses inflammation and oxidative stress induced by subarachnoid hemorrhage [51].

In the present study, the sperm acrosome integrity in frozen sperm with gentiobiose and frozen sperm with trehalose was higher than in frozen control. It may be attributed to the inhibiting effect of freezing medium supplemented with trehalose or gentiobiose on ROS production during cryopreservation. The low or controlled concentrations of ROS play an essential role in sperm physiological processes, including capacitation and acrosome reaction [52, 53]. Therefore, it is proposed that gentiobiose and trehalose probably increase the acrosome integrity by reducing ROS.

DNA fragmentation in sperm occurs during cryopreservation because of apoptosis. Moreover, oxidative stress and nucleophilic attack of free radicals on DNA lead to this fragmentation during cryopreservation [22, 54]. This study shows that adding a gentiobiose or trehalose to the sperm freezing medium increases the total antioxidant capacity, improving the survival of cryopreserved sperm and DNA integrity. This data is consistent with the previous studies, which indicate the positive effect of trehalose and gentiobiose on sperm DNA integrity [55–57]. Furthermore, for the preservation of biomaterials, disaccharides such as trehalose and gentiobiose are more effective than monosaccharides like glucose [58, 59] because a higher glass transition temperature is observed in trehalose or gentiobiose than that in monosaccharides, which results in increased stability of biomaterials stored with these components in less desirable conditions [60, 61].

Annexin V is a calcium-dependent phospholipid-binding protein that binds to phosphatidylserine (PS) and identifies apoptotic cells by binding to exposed PS. When PS translocates from the internal surface of the cell membrane to the external surface, it is a biomarker of cellular apoptosis. Thus sperm stained with FITC is considered to be apoptotic. Sperm cryopreservation causes the production of superoxide anion, and hydrogen peroxidase leads to an increase in the level of cytochrome C in mitochondria, which can activate the mitochondrial or internal path of apoptosis [62–64]. Mitochondria play a crucial role in controlling and creating apoptosis. Apoptosis can also significantly cause frostbite during sperm cryopreservation [28, 65, 66]. Loss of sperm quality during cryopreservation seems to induce apoptosis. Our results found that trehalose and gentiobiose reduce cell apoptosis during cryopreservation. The expression enhancement of BCl2 as the anti-apoptotic gene in the presence of Crocin significantly decreased BAX (a pro-apoptotic gene), demonstrating that adding Crocin to a freezing medium reduced cell apoptosis during apoptosis cryopreservation [67]. Trehalose reduced apoptosis and increased the autophagy and survival levels of the cells against H_2_O_2_ [68]. Trehalose also significantly improves viability, decreases apoptosis, and reduces oxidative stress [69, 70].

The high levels of endogenous ROS in mitochondria can disrupt the mitochondrial respiratory chain and cause programmed cell death [71]. Mitochondrial membrane potential in the current study was increased in the presence of trehalose and gentiobiose during cryopreservation, which is consistent with previous studies. Trehalose could effectively interfere with the magnesium-induced mitochondrial dysfunction in a mouse model of manganism, and trehalose pre-treatment reduced oxidative damage and increased the activation of mitophagy [72]. Trehalose could ameliorate oxidative stress-mediated mitochondrial membrane potential collapse, ATP level decrease, and upregulation of proteins involved in mitochondria stress-related apoptosis pathway [73]. However, no report has been observed on the effect of gentiobiose on mitochondrial membrane potential.

Trehalose and gentiobiose are two sugars in this study that are structurally similar. A comparison of these sugars shows that both have the same number of hydroxyl and hydroxymethyl groups (8 groups), and all of these groups are oriented cylindrically around two pyranose rings (With a slight difference; trehalose has two hydroxymethyl groups, while gentiobiose has only one hydroxymethyl group), and the same number of rings (2 rings) as well as the same number of glycosidic oxygen atoms (1 atom). However, the significant difference between the two sugars is the presence of an extra dihedral angle during the glycosidic binding of gentiobiose to trehalose. Trehalose is a non-reducing disaccharide with alpha-glycosidic binding (αGlc (1→1) αGlc). Gentiobiose is beta-anomeric and is a reducing disaccharide with beta-glycosidic binding (βGlc (1→6) βGlc). This difference has a substantial effect on flexibility. The differences observed in the simulations performed in previous experiments show that trehalose and gentiobiose on the lipid bilayer can be related mainly to the same difference in intrinsic flexibility between the two sugars [31].

The high cryopreservation effects of trehalose and gentiobiose compared to other disaccharides and monosaccharides is related to their structure. Trehalose and gentiobiose can make strong hydrogen bonds with polar groups of biomaterials, thus replacing the water molecules at the membrane-liquid interface and keeping the headgroups at their hydrated position during air drying or freeze-drying [74]. These disaccharides inhibit the biomembrane’s phase transition from lamellar to gel phase and the accompanying leakage [74, 75]. Also, trehalose and gentiobiose have more cryopreservation strength than other sugars such as sucrose and glucose due to the presence of ɑ,ɑ- (1→1) glycosidic binding. Studies have shown that ɑ,ɑ- trehalose stabilizes membranes by binding to cell membrane lipids [76, 77], thereby increasing cell viability, maintaining membrane integrity, and reducing damage caused by the cryopreservation [76, 78]. In addition, previous studies showed that 50 mM of trehalose has better post-thawed sperm parameters than 50 mM sucrose used in the freezing medium at the same concentrations. These beneficial effects of trehalose rather than sucrose have also been reported in animal studies [77, 79]. A comparison of raffinose and trehalose in similar concentrations showed trehalose to give significantly better recovery of intact cells [77].

The current study suggests that gentiobiose could be a better choice due to its unique properties than trehalose during sperm cryopreservation. There are four significant functional differences between these two sugars; 1) There is a higher rate of residual bilayer coating in the presence of trehalose than in gentiobiose. 2) Higher levels of sugar-sugar hydrogen bonds (sugar clustering) are observed in the presence of trehalose than gentiobiose. 3) Fewer glucose-lipid hydrogen bonds are observed in the presence of trehalose than in gentiobiose. In other words, gentiobiose easily attaches to the membrane. Therefore, hydration of the bilayer membrane occurs less in the presence of gentiobiose and minor damage to the cell during the freezing process [31, 80]. 4) The penetration depth of trehalose into the bilayer membrane is less than that of gentiobiose due to the hydrogen bonds that gentiobiose form with esters (such as sphingolipids, phosphatidylcholine, phosphatidylethanolamine, ceramides, etc.). These observations may indirectly have related to the specific conformational characteristics of the trehalose, including a high tendency to self-aggregation, high hydrophilic strength, and a lack of flexibility [31].

## Conclusion

Sperm viability and motion parameters were enhanced by adding trehalose or gentiobiose to the cryomedia. Further evaluation of the molecular profile of sperm during cryopreservation using these disaccharides showed a remarkable reduction in the oxidative damage to DNA and membrane lipids of sperm. Gentiobiose considerably improved sperm motion parameters, DNA and membrane integrity, and mitochondrial membrane potential in post-thawed sperm compared to those stored in conventional media with no additives or in the presence of trehalose as cryoprotectant. The current results showed the potential advantages of using gentiobiose as a protecting agent alongside existing compounds used in cryopreservation. Further studies should use the present results on sperm and illustrate the targeted pathways of cryo-injury and the modulatory impressions of trehalose and gentiobiose.

## Supporting information

S1 File Contains all the supporting tables. (PDF)

S2 File (RAR)

S3 File (RAR)

S4 File (RAR)

S5 File (RAR)

S6 File (RAR)

## Abbreviations

HSA: human serum albumin
CASA: computer-assisted sperm analysis
VCL: curvilinear velocity
VSL: straight linear velocity
VAP: average path velocity
LIN: linearity
STR: straightness
ALH: amplitude of lateral head displacement
BCF: beat-cross frequency
WHO: world health organization
ROS: reactive oxygen species
MDA: malondialdehyde
TBA: thiobarbituric acid
TAC: total antioxidant capacity
HOS: hypo-osmotic swelling
FITC-PSA: fluorescein conjugated lectin Pisum sativum agglutinin
JC-1: 5,5′,6,6′-tetrachloro-1,1′3,3′-tetrathylbenzimidazolyl-carbocyanine iodide
AOT: acridine orange tes
DFI: DNA fragmentation index
MMP: mitochondrial membrane potential
PI: propidium iodide
An: Annexin
