# Supplementary material for: Beneficial effects of trehalose and gentiobiose on human sperm cryopreservation
Source: PLoS One. 2023 Apr 13;18(4):e0271210. doi: 10.1371/journal.pone.0271210 (PMC10101468; doi:10.1371/journal.pone.0271210)
Supplement: S1 File — (PDF) [file pone.0271210.s001.pdf]

Beneficial effects of trehalose and gentiobiose on human sperm cryopreservation

Dariush Gholami[1,2,3*], Mohsen Sharafi[4], Vahid Esmaeili[3], Touba Nadri[5]. Loghman Alaei[6], Gholamhossein Riazi[1*], Abdolhossein Shahverdi[3]*

[1] Institute of Biochemistry and Biophysics (IBB), University of Tehran, Tehran, Iran

[2] Faculty of Biotechnology, Amol University of Special Modern Technologies, Amol, Iran

[3] Department of Embryology at Reproduction Biomedicine Research Center, Royan Institute for Reproductive Biomedicine, ACER, Tehran, Iran

[4] Department of Poultry Science, Faculty of Agriculture, Tarbiat Modares University, Tehran, Iran

[5] Department of Animal Science, Faculty of Agriculture, Urmia University, Urmia, Iran

[6] Department of Biological Science, Faculty of Science, University of Kurdistan, Sanandaj, Iran

*** Corresponding authors:**

Dariush Gholami

Faculty of Biotechnology, Amol University of Special Modern Technologies, Amol, Iran. Postal Code: 4615664616, Phone No: (+98 11) 44442137. Fax No: (+98 11) 44154265, Email: d.gholami@ausmt.ac.ir

Gholamhossein Riazi

Institute of Biochemistry and Biophysics, University of Tehran, P.O. Box: 13145-1365, Tehran, Iran. Postal Code: 1417614335, Phone No: +98 21 66956981. Fax No: +98 21 66404680, Email: ghriazi@ut.ac.ir

Abdolhossein Shahverdi

Royan Institute for Reproductive Biomedicine. No.2, Hafez St., Banihashem St., Resalat Ave., Tehran, Iran; P.O.Box: 16635-148; Tel: +98-21-22339940; Fax: +98-21-23562677, Email: shahverdi@royaninstitute.org

S1 Table. Descriptive and analysis of variance (ANOVA) of dosage of trehalose and gentiobiose. The concentration of 0.05M of trehalose is presented as the highest total motility among the other concentrations (A). The concentration of 0.05M of gentiobiose is shown as the most increased total motility among the different concentrations (B). Refer to the text for further details. P < 0.05 was regarded as statistically significant.

Descriptives Table A

Total_Motility (%)

					95% Confidence Interval for Mean			
	N	Mean	Std. Deviation	Std. Error	Lower Bound	Upper Bound	Minimum	Maximum
5%HSA	25	33.4800	2.00250	.40050	32.6534	34.3066	30.00	37.00
5%HSA + 0.025M trehalose	25	36.4000	2.87228	.57446	35.2144	37.5856	30.00	41.00
5%HSA + 0.05M trehalose	25	48.5200	1.89561	.37912	47.7375	49.3025	45.00	52.00
5%HSA + 0.1M trehalose	25	43.3200	3.67106	.73421	41.8047	44.8353	39.00	49.00
5%HSA + 0.2M trehalose	25	35.0400	2.18861	.43772	34.1366	35.9434	32.00	39.00
Total	125	39.3520	6.25952	.55987	38.2439	40.4601	30.00	52.00

ANOVA Table A

Total_Motility (%)

	Sum of Squares	df	Mean Square	F	Sig.
Between Groups	4039.632	4	1009.908	147.994	.000
Within Groups	818.880	120	6.824		
Total	4858.512	124			

S1 Table. Continued…

Descriptives Table B

Total Motility (%)

	N	Mean	Std. Deviation	Std. Error	95% Confidence Interval for Mean		Minimum	Maximum
					Lower Bound	Upper Bound		
5%HSA	25	33.2400	1.66533	.33307	32.5526	33.9274	30.00	36.00
5%HSA + 0.025M gentiobiose	25	48.5600	1.89473	.37895	47.7779	49.3421	42.00	51.00
5%HSA + 0.05M gentiobiose	25	59.7200	1.42945	.28589	59.1300	60.3100	58.00	63.00
5%HSA + 0.1M gentiobiose	25	51.3600	3.22594	.64519	50.0284	52.6916	41.00	58.00
5%HSA + 0.2M gentiobiose	25	41.4800	1.78232	.35646	40.7443	42.2157	39.00	45.00
Total	125	46.8720	9.24660	.82704	45.2351	48.5089	30.00	63.00

ANOVA Table B

Total Motility (%)

	Sum of Squares	df	Mean Square	F	Sig.
Between Groups	10074.192	4	2518.548	572.658	.000
Within Groups	527.760	120	4.398		
Total	10601.952	124			

S2 Table. Descriptive and analysis of variance (ANOVA) of the CASA analysis. Total and progressive motility and sperm motion variables. $P < 0.05$ was regarded as statistically significant. VCL, curvilinear velocity; VSL, straight linear velocity; VAP, average path velocity; LIN, linearity; STR, straightness; ALH, the amplitude of lateral head displacement; BCF, beat-cross frequency**.** Refer to the text for further details.

Descriptives

						95% Confidence Interval for Mean			
		N	Mean	Std. Deviation	Std. Error	Lower Bound	Upper Bound	Minimum	Maximum
Total_Motility (%)	Fresh Control	25	82.9360	6.13911	1.22782	80.4019	85.4701	75.16	94.46
	Frozen Control	25	46.8484	2.50516	.50103	45.8143	47.8825	41.21	53.28
	Frozen Trehalose	25	58.4452	3.89087	.77817	56.8391	60.0513	50.00	63.48
	Frozen Gentiobiose	25	68.6676	2.85703	.57141	67.4883	69.8469	63.17	73.58
	Total	100	64.2243	13.94221	1.39422	61.4579	66.9907	41.21	94.46
Progressive_Motility (%)	Fresh Control	25	61.3276	11.08915	2.21783	56.7502	65.9050	32.57	80.63
	Frozen Control	25	21.9632	6.56646	1.31329	19.2527	24.6737	13.57	35.89
	Frozen Trehalose	25	30.5064	9.85187	1.97037	26.4397	34.5731	15.00	47.93
	Frozen Gentiobiose	25	41.3536	12.96228	2.59246	36.0030	46.7042	14.81	61.34
	Total	100	38.7877	17.98004	1.79800	35.2201	42.3553	13.57	80.63
Non-Progressive_Motility (%)	Fresh Control	25	22.2348	8.71922	1.74384	18.6357	25.8339	8.79	44.19
	Frozen Control	25	24.8880	6.57238	1.31448	22.1751	27.6009	11.48	35.66
	Frozen Trehalose	25	27.5256	9.75967	1.95193	23.4970	31.5542	12.90	43.43
	Frozen Gentiobiose	25	27.2576	11.71733	2.34347	22.4209	32.0943	10.92	53.91
	Total	100	25.4765	9.48049	.94805	23.5954	27.3576	8.79	53.91
VCL (μm/s)	Fresh Control	25	84.7400	9.99383	1.99877	80.6147	88.8653	69.59	104.14
	Frozen Control	25	49.8448	16.33611	3.26722	43.1016	56.5880	29.29	93.13
	Frozen Trehalose	25	60.2712	19.80885	3.96177	52.0945	68.4479	27.05	88.39
	Frozen Gentiobiose	25	66.1240	17.56268	3.51254	58.8745	73.3735	22.23	92.53
	Total	100	65.2450	20.52107	2.05211	61.1732	69.3168	22.23	104.14
VSL (μm/s)	Fresh Control	25	44.5124	15.80981	3.16196	37.9864	51.0384	11.43	78.60
	Frozen Control	25	22.3088	7.98071	1.59614	19.0145	25.6031	4.91	32.91

	Frozen Trehalose	25	27.1364	10.03703	2.00741	22.9933	31.2795	10.64	45.92
	Frozen Gentiobiose	25	35.6576	12.17921	2.43584	30.6303	40.6849	15.30	55.99
	Total	100	32.4038	14.45184	1.44518	29.5362	35.2714	4.91	78.60
VAP (µm/s)	Fresh Control	25	58.7628	18.31893	3.66379	51.2011	66.3245	16.31	89.43
	Frozen Control	25	27.1704	11.43368	2.28674	22.4508	31.8900	10.23	45.39
	Frozen Trehalose	25	33.0076	13.11111	2.62222	27.5956	38.4196	13.51	50.76
	Frozen Gentiobiose	25	46.1532	10.51469	2.10294	41.8129	50.4935	28.12	63.10
	Total	100	41.2735	18.22787	1.82279	37.6567	44.8903	10.23	89.43
LIN (%)	Fresh Control	25	49.3836	8.89844	1.77969	45.7105	53.0567	33.64	73.19
	Frozen Control	25	32.0816	14.06917	2.81383	26.2741	37.8891	15.16	59.83
	Frozen Trehalose	25	40.2088	13.90198	2.78040	34.4703	45.9473	8.54	68.01
	Frozen Gentiobiose	25	46.1368	7.99341	1.59868	42.8373	49.4363	25.35	63.05
	Total	100	41.9527	13.16290	1.31629	39.3409	44.5645	8.54	73.19
STR (%)	Fresh Control	25	74.5044	6.90946	1.38189	71.6523	77.3565	57.80	87.89
	Frozen Control	25	64.0208	14.94747	2.98949	57.8508	70.1908	39.94	84.11
	Frozen Trehalose	25	67.5844	14.57920	2.91584	61.5664	73.6024	25.97	89.87
	Frozen Gentiobiose	25	74.3620	6.93398	1.38680	71.4998	77.2242	54.12	86.07
	Total	100	70.1179	12.22036	1.22204	67.6931	72.5427	25.97	89.87
ALH (µm/s)	Fresh Control	25	2.3648	.60301	.12060	2.1159	2.6137	1.07	3.32
	Frozen Control	25	1.5896	.56089	.11218	1.3581	1.8211	.56	2.93
	Frozen Trehalose	25	1.7656	.43082	.08616	1.5878	1.9434	.71	2.66
	Frozen Gentiobiose	25	2.0772	.41425	.08285	1.9062	2.2482	.92	2.87
	Total	100	1.9493	.58304	.05830	1.8336	2.0650	.56	3.32
BCF (µm/s)	Fresh Control	25	15.2596	4.27037	.85407	13.4969	17.0223	1.21	18.63
	Frozen Control	25	9.6776	2.34158	.46832	8.7110	10.6442	6.40	14.60
	Frozen Trehalose	25	10.1368	3.20511	.64102	8.8138	11.4598	6.07	20.00
	Frozen Gentiobiose	25	12.4412	1.24425	.24885	11.9276	12.9548	9.72	15.37
	Total	100	11.8788	3.68399	.36840	11.1478	12.6098	1.21	20.00

S2 Table. Continued…

ANOVA

		Sum of Squares	df	Mean Square	F	Sig.
Total_Motility (%)	Between Groups	17629.763	3	5876.588	349.454	.000
	Within Groups	1614.384	96	16.817		
	Total	19244.147	99			
Progressive_Motility (%)	Between Groups	21656.867	3	7218.956	66.971	.000
	Within Groups	10348.028	96	107.792		
	Total	32004.895	99			
Non-Progressive_Motility (%)	Between Groups	455.652	3	151.884	1.727	.167
	Within Groups	8442.432	96	87.942		
	Total	8898.084	99			
VCL (µm/s)	Between Groups	16068.313	3	5356.104	20.068	.000
	Within Groups	25622.000	96	266.896		
	Total	41690.313	99			
VSL (µm/s)	Between Groups	7171.498	3	2390.499	16.993	.000
	Within Groups	13505.205	96	140.679		
	Total	20676.704	99			
VAP (µm/s)	Between Groups	14922.740	3	4974.247	26.573	.000
	Within Groups	17970.533	96	187.193		
	Total	32893.273	99			
LIN (%)	Between Groups	4330.119	3	1443.373	10.806	.000
	Within Groups	12822.800	96	133.571		
	Total	17152.919	99			
STR (%)	Between Groups	2021.175	3	673.725	5.068	.003
	Within Groups	12763.219	96	132.950		
	Total	14784.394	99			
ALH (µm/s)	Between Groups	8.803	3	2.934	11.336	.000
	Within Groups	24.850	96	.259		

	Total	33.653	99			
BCF (μm/s)	Between Groups	490.649	3	163.550	18.407	.000
	Within Groups	852.959	96	8.885		
	Total	1343.608	99			

S3 Table. Descriptive and analysis of variance (ANOVA) of the sperm viability using the Eosin-Nigrosin staining. The percentage of viable cells differed in all experimental groups. $P < 0.05$ was regarded as statistically significant. Refer to the text for further details.

Descriptives

Live cells (%)

					95% Confidence Interval for Mean			
	N	Mean	Std. Deviation	Std. Error	Lower Bound	Upper Bound	Minimum	Maximum
Fresh Control	25	76.1200	3.15331	.63066	74.8184	77.4216	71.00	82.00
Frozen Control	25	41.8800	2.89137	.57827	40.6865	43.0735	37.00	48.00
Frozen Trehalose	25	59.8000	1.29099	.25820	59.2671	60.3329	58.00	62.00
Frozen Gentiobiose	25	67.8000	1.44338	.28868	67.2042	68.3958	65.00	71.00
Total	100	61.4000	12.93340	1.29334	58.8337	63.9663	37.00	82.00

ANOVA

Live cells (%)

	Sum of Squares	df	Mean Square	F	Sig.
Between Groups	16030.720	3	5343.573	969.209	.000
Within Groups	529.280	96	5.513		
Total	16560.000	99			

S4 Table. Descriptive and analysis of variance (ANOVA) of the sperm morphology. Normal and abnormal morphology of sperm as indicated by Papanicolaou staining. the percentage of abnormal morphology differs in all of the groups. P < 0.05 was regarded as statistically significant. Refer to the text for further details.

Descriptives

Abnormal Morphology (%)

	N	Mean	Std. Deviation	Std. Error	95% Confidence Interval for Mean		Minimum	Maximum
					Lower Bound	Upper Bound		
Fresh Control	25	85.7200	4.19841	.83968	83.9870	87.4530	81.00	94.00
Frozen Control	25	97.0800	3.06757	.61351	95.8138	98.3462	90.00	100.00
Frozen Trehalose	25	93.3200	3.18486	.63697	92.0054	94.6346	84.00	97.00
Frozen Gentiobiose	25	87.6400	4.09145	.81829	85.9511	89.3289	81.00	95.00
Total	100	90.9400	5.80111	.58011	89.7889	92.0911	81.00	100.00

ANOVA

Abnormal Morphology (%)

	Sum of Squares	df	Mean Square	F	Sig.
Between Groups	2037.560	3	679.187	50.385	.000
Within Groups	1294.080	96	13.480		
Total	3331.640	99			

S5 Table. Descriptive and analysis of variance (ANOVA) of the membrane integrity of sperm was estimated using the HOS test. The percentage of membrane-intact differs among the experimental groups (B). $P < 0.05$ was regarded as statistically significant. Refer to the text for further details.

Descriptives

HOST (%)

					95% Confidence Interval for Mean			
	N	Mean	Std. Deviation	Std. Error	Lower Bound	Upper Bound	Minimum	Maximum
Fresh Control	25	88.2400	1.71464	.34293	87.5322	88.9478	84.00	91.00
Frozen Control	25	36.7600	2.86182	.57236	35.5787	37.9413	31.00	43.00
Frozen Trehalose	25	64.4000	4.93288	.98658	62.3638	66.4362	54.00	73.00
Frozen Gentiobiose	25	75.3600	3.05341	.61068	74.0996	76.6204	70.00	80.00
Total	100	66.1900	19.34936	1.93494	62.3507	70.0293	31.00	91.00

ANOVA

HOST (%)

	Sum of Squares	df	Mean Square	F	Sig.
Between Groups	35990.510	3	11996.837	1071.465	.000
Within Groups	1074.880	96	11.197		
Total	37065.390	99			

S6 Table. Descriptive and analysis of variance (ANOVA) of the acrosome integrity was assessed using the fluorescein conjugated lectin Pisum sativum agglutinin (FITC-PSA) staining. $P < 0.05$ was regarded as statistically significant. Refer to the text for further details.

Descriptives

Acrosome_Intact (%)

	N	Mean	Std. Deviation	Std. Error	95% Confidence Interval for Mean		Minimum	Maximum
					Lower Bound	Upper Bound		
fresh control	25	94.6600	2.44404	.48881	93.6511	95.6689	90.00	99.00
frozen control	25	54.1920	3.69977	.73995	52.6648	55.7192	49.00	61.00
frozen trehalose	25	66.4000	2.92973	.58595	65.1907	67.6093	61.00	71.00
frozen gentiobiose	25	72.8400	3.51994	.70399	71.3870	74.2930	67.00	79.00
Total	100	72.0230	15.08958	1.50896	69.0289	75.0171	49.00	99.00

ANOVA

Acrosome_Intact (%)

	Sum of Squares	df	Mean Square	F	Sig.
Between Groups	21566.599	3	7188.866	707.654	.000
Within Groups	975.238	96	10.159		
Total	22541.837	99			

S7 Table. Descriptive and analysis of variance (ANOVA) of the DNA fragmentation index was determined using AOT. Intact DNA is shown as green cells, and damaged DNA is observed as yellow and red cells by fluorescent microscopy with 100× magnification. The DFI was significantly different in all of the groups. $P < 0.05$ was regarded as statistically significant. Refer to the text for further details.

Descriptives

DFI (%)

	N	Mean	Std. Deviation	Std. Error	95% Confidence Interval for Mean		Minimum	Maximum
					Lower Bound	Upper Bound		
Fresh Control	25	6.4048	.92091	.18418	6.0247	6.7849	5.00	8.00
Frozen Control	25	17.1876	1.87620	.37524	16.4131	17.9621	13.00	20.00
Frozen Trehalose	25	11.7760	1.56079	.31216	11.1317	12.4203	9.00	14.00
Frozen Gentiobiose	25	8.0680	1.16859	.23372	7.5856	8.5504	6.00	10.00
Total	100	10.8591	4.39136	.43914	9.9878	11.7304	5.00	20.00

ANOVA

DFI (%)

	Sum of Squares	df	Mean Square	F	Sig.
Between Groups	1713.041	3	571.014	279.571	.000
Within Groups	196.076	96	2.042		
Total	1909.118	99			

S8 Table. Descriptive and analysis of variance (ANOVA) of the Comparison of MDA, ROS, TAC and mitochondrial membrane potential among the experimental groups. P < 0.05 was regarded as statistically significant. Refer to the text for further details.

Descriptives

		N	Mean	Std. Deviation	Std. Error	95% Confidence Interval for Mean Lower Bound	95% Confidence Interval for Mean Upper Bound	Minimum	Maximum
Seminal_MDA	Fresh Control	25	31.3120	1.94707	.38941	30.5083	32.1157	27.40	34.00
	Frozen Control	25	45.7880	3.34531	.66906	44.4071	47.1689	39.80	50.20
	Frozen Trehalose	25	35.7840	2.70627	.54125	34.6669	36.9011	31.00	41.00
	Frozen Gentiobiose	25	34.1240	2.43743	.48749	33.1179	35.1301	29.50	39.00
	Total	100	36.7520	6.07619	.60762	35.5464	37.9576	27.40	50.20
ROS (mIU/ml)	Fresh Control	25	532.6056	23.87722	4.77544	522.7496	542.4616	500.00	600.00
	Frozen Control	25	1107.7484	60.16344	12.03269	1082.9142	1132.5826	1000.00	1213.00
	Frozen Trehalose	25	838.6448	33.07789	6.61558	824.9909	852.2987	798.00	900.00
	Frozen Gentiobiose	25	751.5600	73.82585	14.76517	721.0862	782.0338	625.00	900.00
	Total	100	807.6397	213.27179	21.32718	765.3219	849.9575	500.00	1213.00
TAC (nmol/μl)	Fresh Control	25	19.1344	.76763	.15353	18.8175	19.4513	17.56	20.00
	Frozen Control	25	5.4200	.82209	.16442	5.0807	5.7593	4.00	7.20
	Frozen Trehalose	25	13.6208	.80207	.16041	13.2897	13.9519	12.30	15.20
	Frozen Gentiobiose	25	14.3024	.52859	.10572	14.0842	14.5206	13.50	15.20
	Total	100	13.1194	5.00536	.50054	12.1262	14.1126	4.00	20.00
red/green	Fresh Control	25	1.9749	.09749	.01950	1.9346	2.0151	1.79	2.14
	Frozen Control	25	.3957	.02326	.00465	.3861	.4053	.36	.44
	Frozen Trehalose	25	.6803	.05764	.01153	.6565	.7041	.56	.79
	Frozen Gentiobiose	25	1.3506	.15908	.03182	1.2849	1.4162	1.04	1.66
	Total	100	1.1003	.62312	.06231	.9767	1.2240	.36	2.14

S8 Table. Continued…

ANOVA

		Sum of Squares	df	Mean Square	F	Sig.
Seminal_MDA	Between Groups	2977.158	3	992.386	140.529	.000
	Within Groups	677.932	96	7.062		
	Total	3655.090	99			
ROS (mIU/ml)	Between Groups	4245380.924	3	1415126.975	527.336	.000
	Within Groups	257619.956	96	2683.541		
	Total	4503000.880	99			
TAC (nmol/µl)	Between Groups	2427.797	3	809.266	1479.590	.000
	Within Groups	52.507	96	.547		
	Total	2480.304	99			
red/green	Between Groups	37.512	3	12.504	1293.269	.000
	Within Groups	.928	96	.010		
	Total	38.440	99			

S9 Table. Descriptive and analysis of variance (ANOVA) of the Sperm staining by Annexin V and PI. An^{-}/PI^{-}, live cells; An^{+}/PI^{-}, early apoptotic cells; An^{+}/PI^{+}, late apoptotic cells; An^{-}/PI^{+}, necrotic cells. $P < 0.05$ was regarded as statistically significant. Refer to the text for further details.

Descriptives

		N	Mean	Std. Deviation	Std. Error	95% Confidence Interval for Mean		Minimum	Maximum
						Lower Bound	Upper Bound		
Annexin V-/PI- (%)	Fresh Control	25	59.5132	2.33160	.46632	58.5508	60.4756	52.50	63.35
	Frozen Control	25	35.0304	2.23496	.44699	34.1079	35.9529	30.24	38.75
	Frozen Trehalose	25	51.6504	1.64824	.32965	50.9700	52.3308	48.60	56.00
	Frozen Gentiobiose	25	57.5072	3.66180	.73236	55.9957	59.0187	50.20	63.00
	Total	100	50.9253	9.99674	.99967	48.9417	52.9089	30.24	63.35
Annexin V+/PI- (%)	Fresh Control	25	12.2784	1.42790	.28558	11.6890	12.8678	10.00	15.34
	Frozen Control	25	15.1320	3.00854	.60171	13.8901	16.3739	5.02	18.52
	Frozen Trehalose	25	11.4784	2.93290	.58658	10.2678	12.6890	2.99	17.20
	Frozen Gentiobiose	25	9.8092	5.15452	1.03090	7.6815	11.9369	4.20	21.30
	Total	100	12.1745	3.86801	.38680	11.4070	12.9420	2.99	21.30
Annexin V+/PI+ (%)	Fresh Control	25	23.5896	3.44723	.68945	22.1667	25.0125	18.30	31.50
	Frozen Control	25	38.1180	2.07399	.41480	37.2619	38.9741	34.25	44.10
	Frozen Trehalose	25	27.2784	1.23562	.24712	26.7684	27.7884	25.34	29.80
	Frozen Gentiobiose	25	23.6568	1.61968	.32394	22.9882	24.3254	21.32	26.80
	Total	100	28.1607	6.36888	.63689	26.8970	29.4244	18.30	44.10
Annexin V-/PI+ (%)	Fresh Control	25	4.6228	2.79971	.55994	3.4671	5.7785	.60	11.30
	Frozen Control	25	12.0064	2.81808	.56362	10.8432	13.1696	7.34	17.80
	Frozen Trehalose	25	9.5884	3.58461	.71692	8.1087	11.0681	4.17	21.40
	Frozen Gentiobiose	25	9.1416	4.00207	.80041	7.4896	10.7936	3.20	15.60
	Total	100	8.8398	4.24389	.42439	7.9977	9.6819	.60	21.40

S9 Table. Continued…

ANOVA

		Sum of Squares	df	Mean Square	F	Sig.
Annexin V-/PI- (%)	Between Groups	9256.176	3	3085.392	464.722	.000
	Within Groups	637.365	96	6.639		
	Total	9893.542	99			
Annexin V+/PI- (%)	Between Groups	370.920	3	123.640	10.691	.000
	Within Groups	1110.269	96	11.565		
	Total	1481.189	99			
Annexin V+/PI+ (%)	Between Groups	3527.659	3	1175.886	231.303	.000
	Within Groups	488.040	96	5.084		
	Total	4015.698	99			
Annexin V-/PI+ (%)	Between Groups	711.548	3	237.183	21.250	.000
	Within Groups	1071.503	96	11.161		
	Total	1783.051	99			
